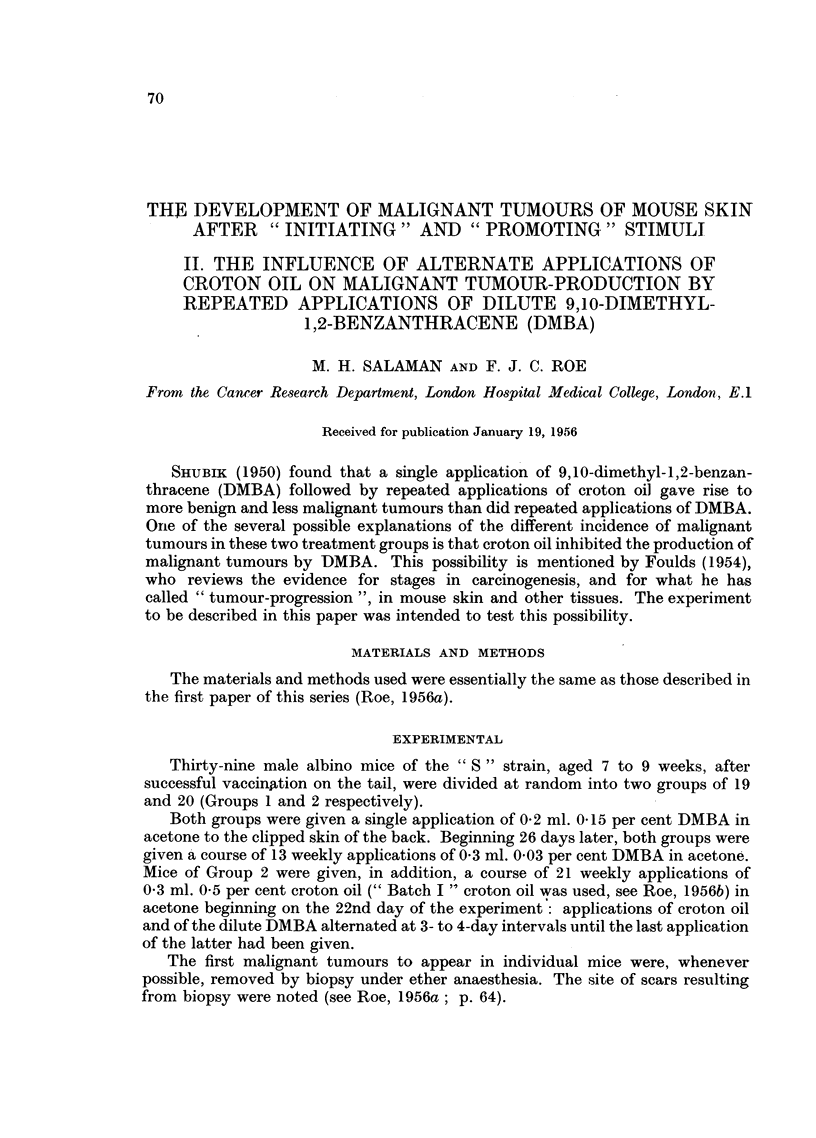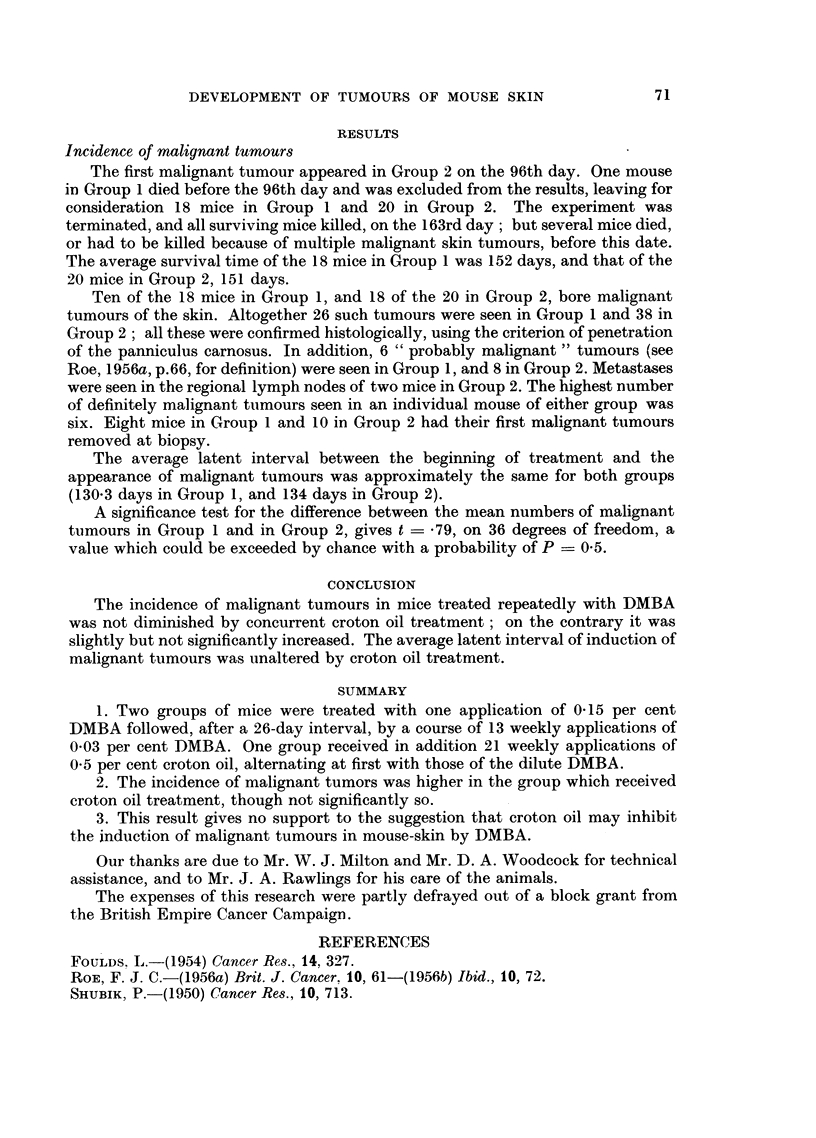# The Development of Malignant Tumours of Mouse Skin after “Initiating” and “Promoting” Stimuli. II. The Influence of Alternate Applications of Croton Oil on Malignant Tumour-production by Repeated Applications of Dilute 9, 10-Dimethyl-1, 2-Benzanthracene (DMBA)

**DOI:** 10.1038/bjc.1956.10

**Published:** 1956-03

**Authors:** M. H. Salaman, F. J. C. Roe


### RESULTS

### *Incidence of malignant tumours*

The first malignant tumour appeared in Group 2 on the 96th day. One mouse in Group 1 died before the 96th day and was excluded from the results, leaving for consideration 18 mice in Group 1 and 20 in Group 2. The experiment was terminated, and all surviving mice killed, on the 163rd day ; but several mice died, or had to be killed because of multiple malignant skin tumours, before this date. The average survival time of the 18 mice in Group 1 was 152 days, and that of the 20 mice in Group 2, 151 days.

Ten of the 18 mice in Group 1, and 18 of the 20 in Group 2, bore malignant tumours of the skin. Altogether 26 such tumours were seen in Group 1 and 38 in Group 2 ; all these were confirmed histologically, using the criterion of penetration of the panniculus carnosus. In addition, 6 " probably malignant " tumours (see Roe, 1956*a*, p.66, for definition) were seen in Group 1, and 8 in Group 2. Metastases were seen in the regional lymph nodes of two mice in Group 2. The highest number of definitely malignant tumours seen in an individual mouse of either group was six. Eight mice in Group 1 and 10 in Group 2 had their first malignant tumours removed at biopsy.

The average latent interval between the beginning of treatment and the appearance of malignant tumours was approximately the same for both groups (130·3 days in Group 1, and 134 days in Group 2).

A significance test for the difference between the mean numbers of malignant tumours in Group 1 and in Group 2, gives $t = \cdot 79$, on 36 degrees of freedom, a value which could be exceeded by chance with a probability of $P = 0{\cdot}5$.

### CONCLUSION

The incidence of malignant tumours in mice treated repeatedly with DMBA was not diminished by concurrent croton oil treatment ; on the contrary it was slightly but not significantly increased. The average latent interval of induction of malignant tumours was unaltered by croton oil treatment.

### SUMMARY

1. Two groups of mice were treated with one application of 0·15 per cent DMBA followed, after a 26-day interval, by a course of 13 weekly applications of 0·03 per cent DMBA. One group received in addition 21 weekly applications of 0·5 per cent croton oil, alternating at first with those of the dilute DMBA.

2. The incidence of malignant tumors was higher in the group which received croton oil treatment, though not significantly so.

3. This result gives no support to the suggestion that croton oil may inhibit the induction of malignant tumours in mouse-skin by DMBA.

Our thanks are due to Mr. W. J. Milton and Mr. D. A. Woodcock for technical assistance, and to Mr. J. A. Rawlings for his care of the animals.

The expenses of this research were partly defrayed out of a block grant from the British Empire Cancer Campaign.

### REFERENCES

FOULDS, L.—(1954) *Cancer Res.*, **14**, 327.
ROE, F. J. C.—(1956*a*) *Brit. J. Cancer*, **10**, 61—(1956*b*) *Ibid.*, **10**, 72.
SHUBIK, P.—(1950) *Cancer Res.*, **10**, 713.